# Soil organic carbon assessment in perennial agriculture — a base study of Kernza in Alnarp, Sweden

**DOI:** 10.1007/s10661-026-15650-1

**Published:** 2026-07-10

**Authors:** Jonas Ardö, Maja Holm, Karl Ljung

**Affiliations:** Department of Earth and Environmental Sciences, Lund University, Sölvegatan 12, Lund, S-223 62 Sweden

**Keywords:** Minimal detectable difference, Stratification, Perennial agriculture

## Abstract

Increased soil organic carbon (SOC) sequestration reduces atmospheric CO_2_ and builds soil health. A conversion from annual crops to perennial crops may decrease negative environmental impacts and promote desired ecosystem services. Soil carbon credit systems and environmental monitoring require reliable verification of SOC change. Here we report a baseline survey of SOC for an agricultural study site in southern Sweden comprised of a perennial grain (Kernza), and a traditional rotation of annual crops as reference. We performed a systematic baseline soil sampling and calculated the minimal detectable difference to determine sample requirements for significant verification of SOC sequestration over time. The potential benefits of stratified sampling, based on a satellite-based vegetation index, flow accumulation and soil type, were investigated. Results indicate that 48% of the SOC was located in the upper 30 cm and > 70% in the upper 60 cm. SOC below 1 m soil depth was low. The minimal detectable difference, based on 10 soil samples, was 3.4 mg C g soil^−1^, equivalent to 1530 g C m^−2^ in the upper 30 cm. Stratified sampling showed no difference among strata for vegetation index and flow accumulation. We conclude that the baseline survey of SOC will benefit forthcoming studies of carbon cycling within the study site. The pay back from applying stratified sampling seems low, as this is a rather homogenous study area. The minimal detectable difference approach requires an unreasonable number of soil samples to allow significant and valid verification of differences in SOC sequestration over time.

## Introduction

Agriculture is crucial for feeding the world, employs large numbers of people and contributes substantially to the global economy. Simultaneously, agriculture is causing major environmental problems, including deterioration of crucial soil-, water- and biodiversity-related ecosystem services (Breitburg et al., 2018; Foley et al., 2011; Power, 2010; Ramankutty et al., 2018). These problems are mainly caused by soil erosion (Olsson et al., 2023), nutrient leakage (Devlin & Brodie, 2023; Yadav et al., 2023) and massive use of herbicides (Crews et al., 2018; Olsson et al., 2024). Agriculture also accounts for about one-third of the anthropogenic greenhouse gas (GHG) emissions (CO_2_, CH_4_, N_2_O) to the atmosphere (Frank et al., 2024; IPCC, 2019) and has negative effects on biodiversity (IPBES, 2019).

Climate change, manifested through warming and increasing frequency and magnitude of extreme events, is expected to decrease resilience and sustainability of agriculture (Paustian et al., 2016). Negative impacts on land ecosystems and biodiversity (IPCC, 2019) are likely and may even jeopardize food security (Foley et al., 2011). A changing climate also increases the risk of various crop-related pests and infestations (IPPC Secretariat, 2021; Skendžić et al., 2021), with potential negative effects. In general, climate change is projected to negatively impact agricultural productivity even if some positive effects also are expected (Bezner Kerr et al., 2023; IPCC, 2019). Climate changerelated impacts on agriculture, combined with rising food demand between 30 and 100% in year 2100 (Steensland, 2021; Van Dijk et al., 2021), put additional strain on the environment related to agricultural production (Bezner Kerr et al., 2023). To reduce the environmental impact of agriculture while simultaneously and sustainably produce more food as well as contribute to mitigation of climate change, adaptations are needed. This poses a great challenge.

Mitigation of climate change is urgent (IPCC, 2019) and may, among other methods, include soil organic carbon (SOC) sequestration in agricultural soils (Paustian et al., 2016; Rodrigues et al., 2023). Such sequestration can be effective and promote sustainable farming systems through improving soil properties and soil health (Jansson & Hofmockel, 2020), partly as a by-product to increasing SOC (Paustian et al., 2016). Agricultural soils with annual crops are repeatedly disturbed through soil mechanical management such as ploughing, tilling and harrowing. Management that leaves the soils bare increases the risk of erosion (Feng et al., 2025), nutrient leakage (Devlin & Brodie, 2023; Yadav et al., 2023) and adverse effects of climatic extremes (Olsson et al., 2022), decreasing the capacity for SOC accumulation (Liang et al., 2024; Rodrigues et al., 2023). Mitigation of and adaptation to climate change may hence include increased cultivation of perennial crops that reduce the need for soil disturbances (tilling, harrowing etc.) (Crews et al., 2018; Olsson et al., 2024) and decrease the exposure of soils (Feng et al., 2025).

Cultivation of perennial crops may reduce negative environmental impacts from agriculture (Crews et al., 2018; Olsson et al., 2024). This reduction includes decreasing carbon emissions due to less soil disturbances which also benefit SOC storage via lower rates of decomposition (Paustian et al., 2016). Less mechanical soil management also lowers fossil fuel use, whereas year-round vegetative cover with deep roots decreases nutrient leakage (Culman et al., 2013; Devlin & Brodie, 2023; Yadav et al., 2023) and the need for herbicides after the crop establishment (Summers et al., 2021).

Perennial, deep-rooted crops, such as Kernza (*Thinopyrum intermedium*, Intermediate wheatgrass) and Lucerne (*Medicago sativa*, Alfalfa), sequester more carbon in general compared to annual crops (Chapman et al., 2022; Kätterer & Bolinder, 2023; Peixoto et al., 2022). High net ecosystem carbon balances, ranging from −513 to −121 g C m^−2^ year^−1^ (negative values denote a transfer from the atmosphere) based on eddy covariance measurements in the USA, have been reported for perennial cultivation of Kernza (de Oliveira et al., 2018, 2020). For corresponding annual crops, net ecosystem carbon balances ranged from 80 to 320 g C m^−2^ (maize/soybean rotation), and ∼ − 91 g C m^−2^ to ∼ − 156 g C m^−2^ year^−1^ (soybean) (de Oliveira et al., 2020). Observed annual carbon assimilation may be up to 50% higher than carbon lost to respiration but respiration increase over the years, influencing long-term sink strength (de Oliveira et al., 2020). C sink strength for Kernza is also influenced by nitrogen fertilization, precipitation patterns and harvest regime (de Oliveira et al., 2020).

Kätterer and Bolinder (2023) reviewed the impact of agricultural management on SOC stocks and reported an average difference (0.51 Mg C year^−1^ for the upper 25 cm, this equals 51 g C m^−2^ year^−1^) in mean SOC stock change between annual and perennial crops.

Many perennial grain crops can also be used as forage or for biofuel production, but their grain yield is substantially lower than that of annual crops and extensive cultivation ‘would lead to a reduction in annual grain production’ (Soto-Gómez & Pérez-Rodríguez, 2022), a drawback when replacing annual grain crops with perennial. Additional information on perennial agriculture is available elsewhere (Chapman et al., 2022; Crews et al., 2018; Olsson et al., 2024). Based on the potential benefits of perennial agriculture, we find further investigations of these benefits motivated.

**Fig. 1:**
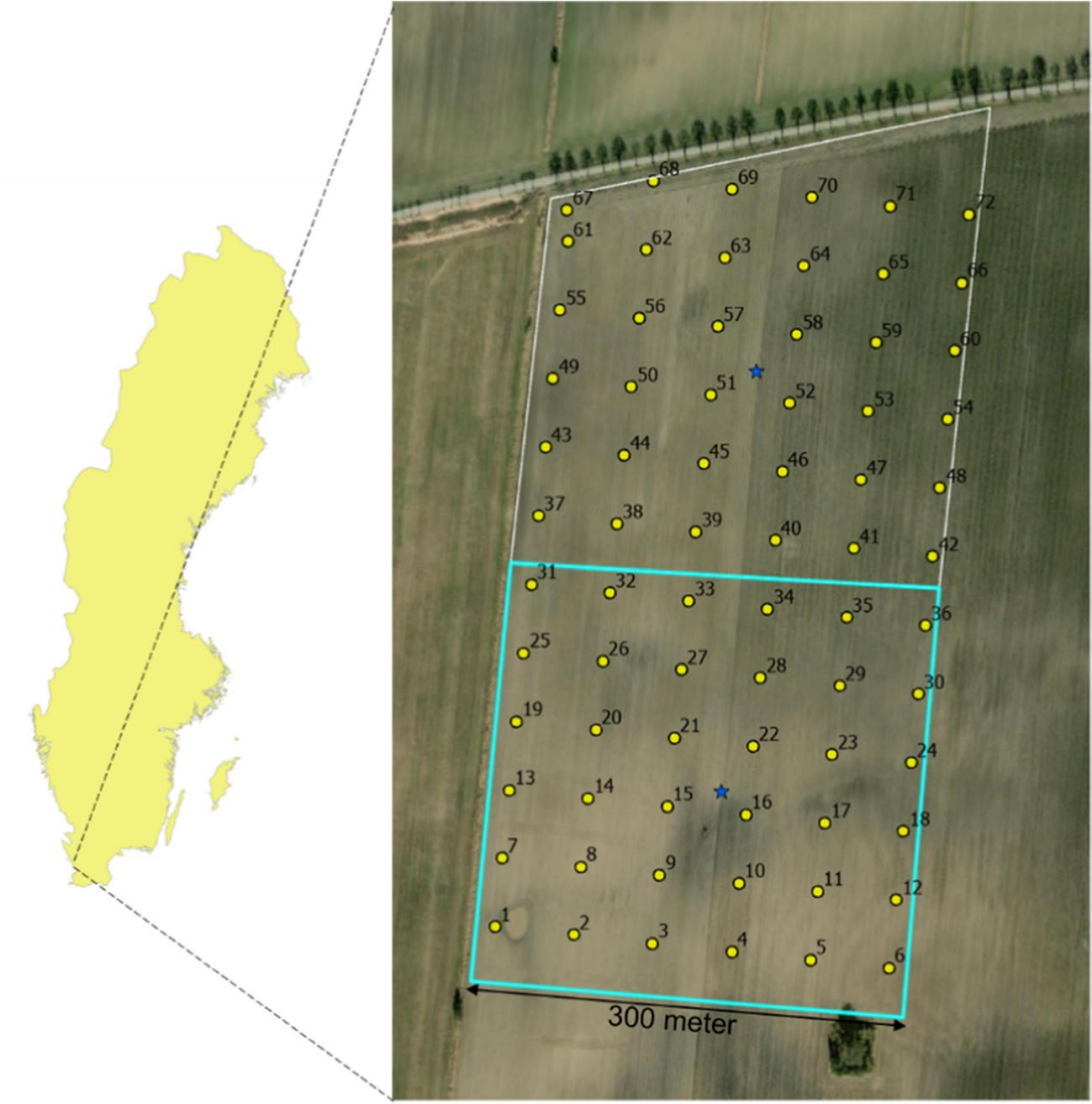
The study area is located at Alnarp in southern Sweden and is approximately 300 by 600 m with Kernza (northern polygon) and control (southern polygon). Blue stars are flux towers and yellow dots are soil sampling locations (*n *= 72)

To investigate potential solutions to some of the agricultural problems mentioned above, a project (PERENNIAL, Olsson & Ardö, 2024) studying a perennial cereal grain was started in 2023, at Alnarp outside Malmö in southern Sweden (Fig. 1). The project aims to investigate and quantify the soil carbon sequestration potential of the perennial grain Kernza® (*Thinopyrum intermedium*, Intermediate wheatgrass). The project focused on soil and vegetation properties related to carbon sequestration, including fluxes of carbon and water which are measured for a 10-ha perennial crop (Kernza) and compared to a 10-ha reference field with a traditional Swedish crop rotation of annual crops (winter wheat, rape seed, sugar beet). The study reported here describes the baseline survey of soil carbon conducted before the project interventions started through the establishment of Kernza and rape seed as a reference crop in September 2023.

Carbon farming, i.e. agricultural practices aimed to sequester SOC and reduce atmospheric GHG concentrations, may be important as means of climate change mitigation (Petropoulos et al., 2025). Via monitoring, reporting and verification, farmers can earn carbon credits. A carbon credit is a certified, tradable carbon offset that is exchanged under a cap and trade system of emissions regulation (Fulwider et al., 2025; Lokuge & Anders, 2022). One carbon credit is normally 1 metric ton (1 Mg C). Methods for monitoring, reporting and verification of the increasing voluntary carbon market vary and are currently discussed (Raffeld et al., 2024; Smith et al., 2020) as they are needed for trustworthy carbon credit systems.

Participation in carbon credit systems requires accurate and efficient verification of sequestration ensuring correct trading. This has resulted in national guidelines for reporting and verification of SOC sequestration, in a range of countries, as recently reviewed (Oldfield et al., 2022; Smith et al., 2020). Studies investigating management effects on soil carbon sequestration also require validation to be able to differentiate useful sequestration methods and environments from less useful methods (Hall, 2022; Kätterer & Bolinder, 2023; Noring et al., 2023). To solve the problem whether if sequestration is trustworthy, several approaches have been proposed (Petropoulos et al., 2025), among other a global soil monitoring, reporting and verification (MRV) platform, based on a combination of measurements and modelling to ensure both representativity and reliability (Smith et al., 2020). These approaches aim to support methods for reliable quantifying of changes in SOC, supporting sound use of carbon credits.

### Aim

A baseline survey of SOC for the comparative experiment in Alnarp is essential to quantify the current SOC stock, and to verify potential changes over time and between the annual and perennial cropping systems in Alnarp. Hence, the aim of this study is to (i) provide baseline quantification of soil organic carbon of the 20-ha area constituting the experimental site, (ii) calculate the minimal detectable difference in SOC to provide an estimate of change required for statistically sound verification of SOC sinks and (iii) investigate the potential benefits of stratified sampling of SOC.

Additionally, we perform and discuss a simple ‘back on the envelope’ upscaling of the SOC sequestration potential via perennial grains in Swedish agriculture and relate it to the GHG emission from Swedish agriculture.

## Material and methods

### Site description

The study site is located in Alnarp, utside Malmö in southern Sweden, where the PERENNIAL project was initiated in 2023 as described above (Olsson & Ardö, 2024) (Fig. 1). The site is flat to gently undulating agricultural area dominated by till and minerogenic soils, mainly postglacial sand, till clay and postglacial clay with small areas of marsh peat (Geological Survey of Sweden, 2018). The site was drained in the 1960 s and has been under agriculture for at least 80 years and probably much longer.

### Soil sampling

On August 15–16, 2023, 72 sampling points in a systematic un-stratified grid (Fig. 1) where sampled and five subsamples of about 100 g were taken at each point using a tractor-mounted, hydraulic Wintex MCL3 soil core sampler (Wintex Agro). The subsamples were linearly spaced 1–2 m apart on an east-west transect with the middle sample at the GPS located sample point. For each sampling point, three depths were sampled (A, 0–30 cm; B, 30–60 cm and C, 60–100 cm) and stored in paper sample bags. The sampling was done after the harvest of the winter wheat but before the seeding of the Kernza and rape seed (*Brassica napus* ssp. *napus*) in the control field. Additional samples were taken down to 200 cm depth at seven randomly chosen sample points (Holm, 2024).

Bulk density was determined at 15 points using an excavator to dig 1 m deep pits which were sampled at 15, 45 and 80 cm depth using the intact core method (FAO, 2020) and 50 mm bulk density cylinders.

### Soil analysis

All soil samples were dried at 40 °C for a week, within 2 days of sampling. All composite samples intended for SOC and soil inorganic carbon (SIC) analysis were then homogenized into a fine powder, grinding them by hand using a mortar and pestle, until all soil aggregates could pass through a 2-mm sieve. Mineral material > 2 mm was put aside after sieving, and both partitions (< 2 mm and > 2 mm) were labelled and weighed. Bulk density samples were weighed in their bags and the mean bag weight was subtracted. SIC was removed through acidification with 1 M hydrochloric acid (HCl) in Ag capsules following Brodie et al. (2011).

Total organic carbon (TOC) and total carbon (TC) content was analysed using a Costech ECS 4010 elemental analyser. The instrument was calibrated every 100–150 samples and for each calibration run, four acetanilide standards (a series of 0.2 mg, 0.5 mg, 1 mg and 2 mg). Two reference soil samples were run after each calibration and the drift was monitored by running an acetanilide sample every tenth sample.

To determine sample representativity, since only 10–20 mg out of the full 300–800 g samples was analysed, 20 replicate subsamples were run on one randomly selected sample (Fig. 8).

The bulk density was calculated using the volume of the cylinder and the weight of the sample. This value was then corrected based on the fine earth fraction (< 2 mm) versus the coarse mineral (> 2 mm) fraction, since the C stock is calculated using the SOC content of the fine earth fraction.

### Stratification

A stratified sampling design may be useful when variability within the strata is minimized and variability among strata is maximized (Donovan, 2013). Simultaneously, the covariate used for stratification should be strongly correlated with SOC. Stratification can reduce the amount of soil samples needed to be able to verify SOC change over time (Potash et al., 2022). To evaluate the performance and applicability of the stratification, a Kruskal-Wallis *H*-test was performed to test for significant differences in SOC among the different strata (MacFarland & Yates, 2016). I.e. the null hypothesis tested is that the median SOC content is the same across included strata. Without significant differences among strata, choosing stratified random over simple random or grid sampling to reduce the required number of samples would be unmotivated, as it requires extra labour.

Three stratifications were tested to investigate if the total number of samples collected could be decreased through a stratification of the sample area. It should be mentioned that the study area is rather homogeneous regarding topography, but with some variability regarding soil types. We stratified the area under investigation using the following covariates: (1) the plant phenology index (PPI), (2) flow accumulation and (3) soil type, as further described below. We tested stratifications using the SOC in the upper layer (A, 0–30 cm). The full 20-ha area (72 samples) were treated together as one field.

#### Plant phenology index

The PPI is derived from Sentinel-2 data and is a proxy for green leaf area index (Jin & Eklundh, 2014). PPI is a vegetation index developed for monitoring vegetation growth, which is better suited for northern areas than NDVI, partly because it takes snow cover into account and due to its almost linear relationship versus green leaf area index (Jin & Eklundh, 2014). Here we use a seasonally integrated measure (the sum of all daily PPI values from the start to the end of the growing season) of relative vegetation productivity (Seasonal Productivity SPROD, (Smets et al., 2023)) with 10-m spatial resolution. We averaged SPROD per grid cell for the 2017–2022 period and used this average for stratification into three strata, low (111.6–135.6, *n *= 15) medium (135.6–159.7, *n *= 40) and high productivity (159.7–183.8, *n *= 18). The null hypothesis tested is that the median SOC content does not differ among PPI strata.

#### Flow accumulation

Flow accumulation is quantified as the number of upstream grid cells flowing into each grid cell after calculating the flow direction in a digital elevation model (DEM) in raster format. We calculated flow accumulation using a multiple flow direction (MFD) method (Qin et al., 2007) and a 2-m spatial resolution DEM from the Swedish national survey (Lantmäteriet, 2019). The resulting flow accumulation was stratified into three strata (*n* sample points per stratum), #1 with a flow accumulation of 0–15 cells (*n *= 43), #2 with 16–30 cells (*n *= 13) and #3 with > 30 cells (*n *= 16) (Fig. 5). Flow accumulation is relevant as areas with higher flow accumulation is assumed to be wetter due to the relatively higher accumulation of water caused by more run-on than run-off. We expect decreasing decomposition of SOC as a results of lower oxygen levels and hence increased accumulation of organic carbon.

#### Soil type

The area was stratified into four strata, based on a soil type in the upper 0.5 m using a digital soil map from the Swedish geological survey (Geological Survey of Sweden, 2018). Included soil types are till clay, postglacial sand, marsh peat and postglacial clay (Fig. 5). Visual, in situ inspection of the soil during sampling showed adequate agreement with the mapped soil types. These soil types may have different SOC content, with till, sand and clay being minerogenic soils while marsh peat is an organogenic soil type. Variability in soil texture (sand, silt and clay content) may also influence the SOC content (Petropoulos et al., 2025).

### Minimal detectable difference

The minimal detectable difference (MDD) is the smallest value (difference) in one-sample hypothesis testing that will reject the null hypothesis (i.e. no difference). It is strongly dependent on the variation (here described by the standard deviation, *σ*) of the samples and calculated (FAO, 2020) as

1 $$\mathrm{M}\mathrm{D}\mathrm{D}=\frac{\sigma }{\sqrt{n}} \cdot ({t}_{\alpha }+ {t}_{\beta })$$

where *σ* = standard deviation, *n *= sample size, *t*_*α*_ = two-tailed inverse of the Student’s *t*-distribution and *t*_*β*_ is the left-tailed inverse of the Student’s *t*-distribution. We calculated MDD with 1 − *β* power and the *α* level of significance where *α* = 0.05 and *β* = 0.90.

### Sequestration potential

Several investigations have reviewed management options promoting SOC sequestration in agriculture (Aronsson et al., 2023; FORMAS, 2021; Hall, 2022; Jordbruksverket, 2010; Jordbruksverket & Naturvårdsverket, 2022); Kätterer & Bolinder, 2023; Noring et al., 2023; Sierra et al., 2024), reporting a variety management options and sequestration rates. We assume potential SOC sequestration rates for perennial crops to range from 30 to 500 g C m^−2^ year^−1^ (de Oliveira et al., 2018, 2020). Based on this, we outline three (low, medium, high) tentative scenarios for potential SOC sequestration through cultivation of perennial crops in Swedish agriculture. Scenarios include low potential, 10,000 ha × 30 g C m^−2^ year^−1^, which is the sequestration rate used by Svensk Kolinlagring (Swedish Carbon Sequestration, SCS) (Svensk Kolinlagring, 2024), medium, 50,000 ha × 100 g C m^−2^ year^−1^ which uses an upper-end sequestration rate as reported by Kätterer and Bolinder (2023) and high, assuming the maximal annual assimilation rate reported by de Oliveira et al. (2018, 2020), i.e. 100,000 ha × 500 g C m^−2^ year^−1^. It should be noted that management aiming at reducing CO_2_ emission from soils may result in emission changes in other greenhouse gases, such as CH_4_ and N_2_O (Basheer et al., 2024).

We relate the results from these scenarios to current GHG emissions from Swedish agriculture. The perennial crops are assumed to replace traditional cereals like wheat, barley, rye and oats. Perennial cereals have substantially lower grain yields than annual cereals (average winter wheat harvest for Sweden 2020–2023 was 6775 kg ha^−1^ (SCB, 2024)) which may influence farmer profit and food security.

In 2023, Swedish agriculture emitted 6.44 million tons (CO_2_e) GHG, mainly originating from CH_4_ and N_2_O (Jordbruksverket & Naturvårdsverket, 2022). This is 0.97 million tons (13%) lower compared to 1990 or a decrease of 0.03 million tons CO_2_e (0.4% year^−1^) annually (Naturvårdsverket, 2024b). This reduction is smaller than in most other sectors like transport, energy, heating and industry. About 39% (equal to 2.53 million tons CO_2_e or 0.68 million tons C) of the 6.44 million tons CO_2_e of agricultural emissions, originate from agricultural lands. The remining originate from animal metabolism (feed digestion) and storage of manure. Agriculture is the largest source of Swedish total emission of methane (CH_4_) and nitrous oxide (N_2_O) (Naturvårdsverket, 2024a).

Sweden is envisioning zero net emission of GHGs by 2045, and negative emissions (i.e. a sink) after that. It is further assumed that by 2030, the emissions should be 63% lower than 1990 and by 2040, 75% lower than they were in 1990 (Naturvårdsverket, 2024b). For agriculture, this means a reduction from 7.41 to 2.74 million ton CO_2_e year^−1^ by 2030. With the three simple scenarios mentioned above, we approximate to what extent cultivation of novel perennial crops can sequester sufficient amounts of carbon to help achieving the Swedish vision of having zero net emission of greenhouse gases by 2045.

## Results

### Bulk density

The bulk density increased with depth (measured at 15 sites and three depths) with an average (± st. dev) BD of 1.49 ± 0.11 (0–30 cm), 1.67 ± 0.15 (30–60 cm) and 1.68 ± 0.13 g cm^−3^ (60–100 cm).

### SOC content

**Fig. 2:**
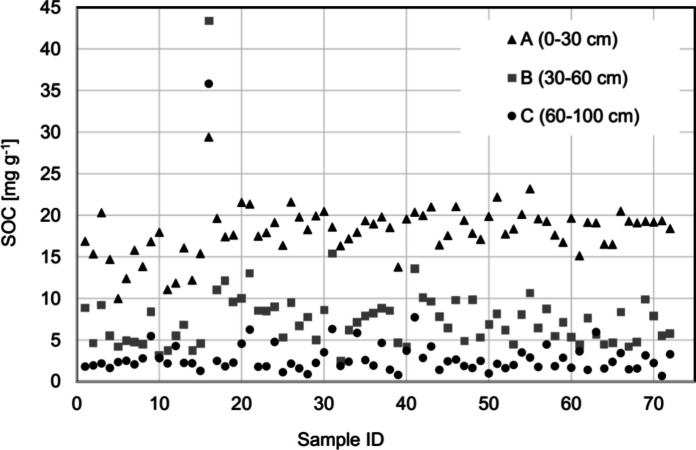
SOC content for 72 sample points (outlined in Fig. 1) at three depths. 10 mg SOC g soil^−1^ equals 15 kg SOC m^−3^ assuming a bulk density of 1.5 g cm^−3^

**Fig. 3:**
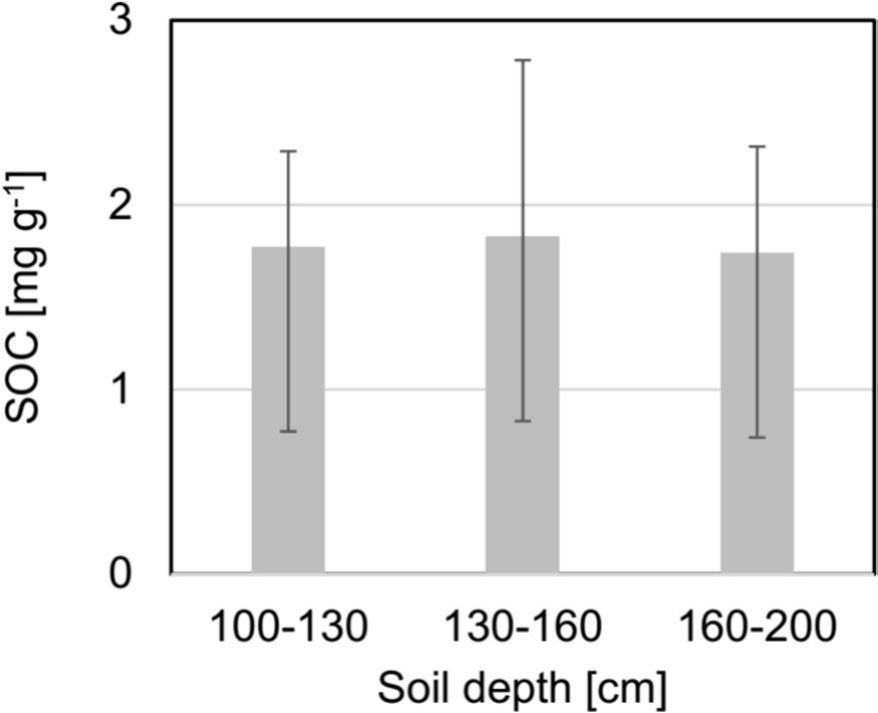
Soil organic carbon content from 100 to 200 cm depth. Error bars are standard deviations, *n* = 15

**Fig. 4:**
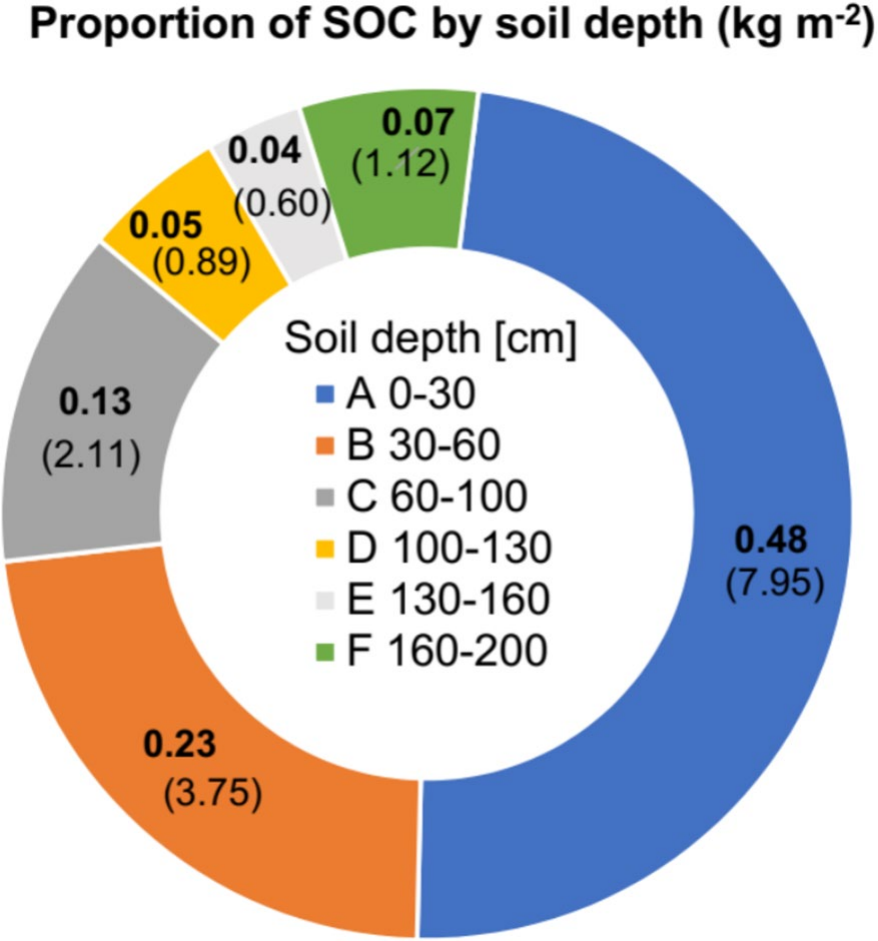
The mean proportion and mass (kg m^−2^) of SOC averaged per depth interval (*n* = 72). On average is 16.4 kg SOC m^−2^ available in the upper two meters with about 50% in the upper 30 cm and about 70% in the upper 60 cm of the soil profile

The SOC content in general decreased with sampling depth and averaged 18.2 (± 0.29) mg g^−1^ soil for A (0–30 cm), 7.7 (± 0.50) mg g^−1^ soil for B (30–60 cm) and 3.1 (± 0.42) mg g^−1^ soil for the C (60–100 cm) layer (Fig. 2). The average SOC content below 1 m was stable (Fig. 3) with 1.78 (± 0.88) mg g^−1^ soil (100–130 cm, *n *= 7), 1.83 (± 1.60) mg g^−1^ soil (130–160, *n *= 7) and 1.74 (± 0.76) mg g^−1^ soil (160–200 cm, *n *= 4). Almost half (48%, 7.95 kg m^−2^) of the SOC is located in the upper 30 cm, > 70% (11.7 kg m^−2^) in the upper 60 cm, and < 30% (4.72 kg m^−2^) from 60 to 200 cm depth (Fig. 4). Sample point #16, located in a depression with organic soil (marsh peat), is a clear outlier with a SOC more than twice compared to all other samples (Fig. 4).

### Stratification

**Fig. 5:**
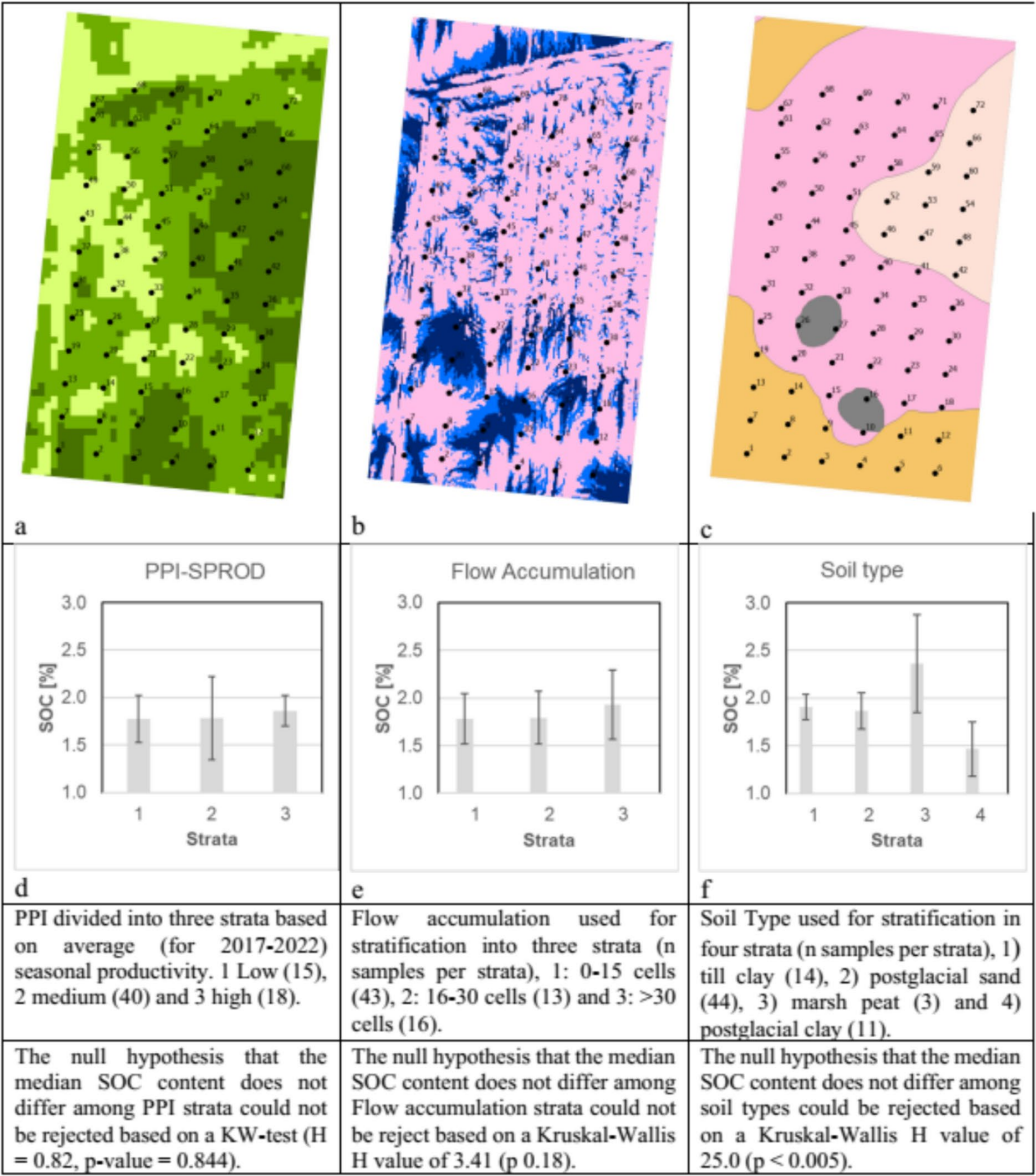
Maps and distribution per stratification using PPI (**a**, **d**), flow accumulation (**b**, **e**) and soil type (**c**, **f**). There are 72 sampling points in total and all are from the A depth (0–30 cm)

For the stratifications using PPI and flow accumulation, the null hypothesis that the median SOC content does not differ among strata, could not be rejected (*p* = 0.84 for PPI and *p *= 0.18 for flow accumulation) (Fig. 5). The stratification using soil type showed a significant difference among median SOC content (*p *< 0.005), supporting a rejection of the null hypothesis that the strata do not differ. This difference is mainly due to the higher SOC content, at all three depths, of one sample point (#16 in Fig. 2 with SOC > 25 mg g^−1^ for all three depths) located on marsh peat with an average SOC content for the stratum of 2.36 mg g^−1^ (median 2.16 mg g^−1^) which can explain this difference among strata for soil type. It should be mentioned that organic soils are normally not target for management options increasing SOC sequestration in agriculture, but rather rewetting options decreasing SOC respiratory losses.

### Minimal detectable difference

**Fig. 6:**
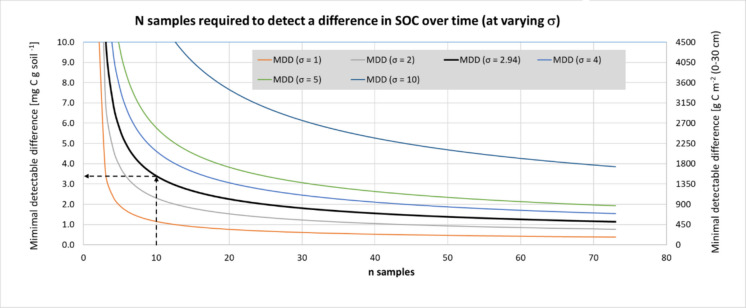
Number of soil samples required to statistically significant detect a difference in soil carbon at different spatial soil C variability (σ). The black bold line represents the variability (σ = 2.94) in soil C content [mg C g soil ^−1^] found in the upper 30 cm in the test site in Alnarp (Fig. 2). The dashed arrows illustrate that 10 soil samples are required to detect a difference of 3.4 mg C g soil^−1^, corresponding to 1530 g C m^−2^ assuming a bulk density of 1.5 [kg dm^−3^]. For the sequestration rates in our three scenarios (30, 100 and 500 g C m^−2^ year^−1^) this corresponds to 51, 15 and 3 years of sequestration before the sequestration could be significantly detected with 10 samples assuming σ = 2.94 mg C g soil^−1^

The MDD (Eq. 1), calculated using the observed standard deviation (*σ* = 2.94 mg C g soil^−1^) for the 0–30-cm level, vary with sample size (Fig. 6). With a sample size of 10, MDD is 3.4 mg C g soil^−1^ (equivalent to 1530 g C m^−2^ in the upper 30 cm). To reach MDD of 1 mg C g soil^−1^ (equivalent to 450 g C m^−2^ in the upper 30 cm) are about 93 samples needed (outside the *n* samples range visible in Fig. 6), ($0.999=\frac{2.94}{\sqrt{93}} \bullet (1.985+ 1.29$) according to Eq. 1). For the sequestration rates used in our three sequestration scenarios (30, 100 and 500 g C m^−2^ year^−1^) this corresponds to 51, 15 and 3 years of sequestration, respectively, before the sequestration could be significantly validated using 10 samples via the MDD approach, assuming *σ* = 2.94 mg C g soil^−1^.

Assuming the soil carbon sequestration rate used by Svensk Kolinlagring (Svensk Kolinlagring, 2024), 300 kg C ha^−1^ year^−1^, equivalent to 30 g C m^−2^ year^−1^ (or an increase of 0.067 mg C g soil^−1^ year^−1^) with a bulk density of 1.5 g cm^−3^. With a project time of 5 years this equals a sequestration of 150 g C m^−2^ for the upper 30 cm of the soil profile. Using the variability observed here (*σ* = 2.94 mg C g soil^−1^) this would require an unrealistic number (~ 800) of soil samples to verify based on the MDD methodology (FAO, 2020; Zar, 1996).

The 20 repeated analyses originating from the same soil sample (point #26A) resulted in a mean C content of 2.31% (23 mg C g soil^−1^) and a standard deviation of 0.16% (1.6 mg C g soil^−1^), a minimum of 1.96% and a maximum of 2.64% (Fig. 8). Hence, analysis also adds some uncertainty.

## Discussion

### SOC

Measurement-based quantification of soil carbon changes requires valid, representative and accurate measurements to properly underpin carbon credits (Smith et al., 2020) and to determine management impact on SOC sequestration (Kätterer & Bolinder, 2023) promoting GHGs mitigation. Here we measured 72 composite samples at three soil depths from 0 to 100 cm (Fig. 2), and 15 samples between 100 and 200 cm soil depth (Fig. 3) to quantify SOC. About 48% of the total (0–2 m depth) SOC is located in the upper 30 cm and > 70% in the upper 60 cm, reasonably in agreement with previous studies (Hiederer, 2009). SOC content below 100 cm is low (1.7 to 1.8 mg C g soil^−1^, i.e. 0.17–0.18%, Fig. 3) and may hence be suitable for SOC sequestration by perennial, deep-rooted plants such as Kernza (De Roissart, 2025; DeHaan et al., 2025) and Lucerne (*Medicago sativa*) (Li et al., 2012). The deep root system of perennial crops provides residues that are less exposed to oxidation and increase root derived SOC (Ledo et al., 2020; Peixoto et al., 2022), partly due to higher microbial biomass and a more active soil microbial community (Li et al., 2025). Subsurface (> 30 cm soil depth) soils, and especially deep soils (> 60 cm), thus offer additional potential for long-term SOC sequestration (Raffeld et al., 2024) and sampling at these levels is required to validate possible vertical redistribution and changes of SOC over time (Button et al., 2022).

Representative data on soil bulk density is also crucial for accurate quantification of SOC, and recent studies (Raffeld et al., 2024; von Haden et al., 2020) recommend the equivalent soil mass (ESM) method over the fixed depth (FD) method. This is especially important when aiming to detect differences including soil bulk density changes over time due to management practices related to tillage, something expected to occur in projects investigating changes from annual crops to perennial crops (Orzech et al., 2021).

Based on the analysed SOC content (*n *= 72, × 3 depths) and bulk density (*n *= 15) of the sampling performed, is the first aim (i), to provide a baseline quantification of SOC, fulfilled.

### MDD

Even with a representative baseline data on SOC and bulk density, it is necessary to validate changes over time, particularly in carbon credit systems. The number of soil samples required for statistically valid verification according to the MDD method (FAO, 2020) is substantial, often approaching 100 and more for short-duration projects or those with low soil carbon sequestration rates (Fig. 6). Collecting and analysing such large sample sets is costly and often deemed impractical, as it may consume a significant portion of the revenue generated from carbon credits. Hence, alternative and more cost-efficient methods for validation of SOC sequestration are needed (Petropoulos et al., 2025), especially in the context of carbon credit programs. We find the second (ii) aim fulfilled, even if the practical applicability of the MDD method can be questioned.

In addition to spatial variability, temporal variability may constitute a substantial proportion of total SOC variability, and this variation is not easily explained by simple environmental drivers alone (Burke et al., 2019; Wuest & Durfee, 2024). Because repeated soil sampling over time is uncommon, such temporal effects are often not fully accounted for, even though they may exceed management effects in magnitude (Wuest & Durfee, 2024). The MDDs reported here may therefore be underestimated. Complementary to the SOC measurements performed at the Alnarp site (Fig. 1), measurements of CO_2_ and CH_4_ fluxes provide information on short-term variation in carbon source–sink dynamics (Widengren et al., 2026). Although flux measurements do not directly quantify changes in SOC pools, they can identify periods of net carbon uptake or loss and thereby provide supporting evidence for expected seasonal to annual changes in soil carbon balance. This distinction is important because net ecosystem carbon balance depends on multiple carbon inputs and outputs, including CO_2_ exchange, CH_4_ emissions, harvested biomass removal and lateral carbon transfers (Chapin et al., 2006). Thus, the flux measurements at Alnarp provide useful contextual information on temporal variability in source–sink strength (Widengren et al., 2026), while SOC stock changes must still be assessed through repeated soil measurements.

### Stratification

Spatial variability of SOC can be high (Conant & Paustian, 2002) and may be strongly related to variables describing plant productivity, such as soil properties, PPI, or related to variables decreasing SOC decomposition rates, such as wet, low oxygen conditions, here represented by flow accumulation area (Fig. 5). Neither PPI nor flow accumulation were suitable covariates for stratification whereas soil type indicates some usefulness in this case. As extensive soil sampling and analysis is time consuming and costly, investigating spatial covariates is motivated to avoid biased or less representative sampling. This motivation may be stronger in areas with larger spatial variability (in soil type, topography, land use history etc.) (Donovan, 2013), than in this study, especially in cases involving organogenic soils. Organogenic soils are normally excluded from agricultural SOC sequestration programs and rather used during rewetting (Günther et al., 2020). This fulfils aim (iii), investigating potential benefits of stratified sampling, which was shown to be low in this study (Fig. 5).

### Sequestration potential of Swedish farmlands

**Fig. 7:**
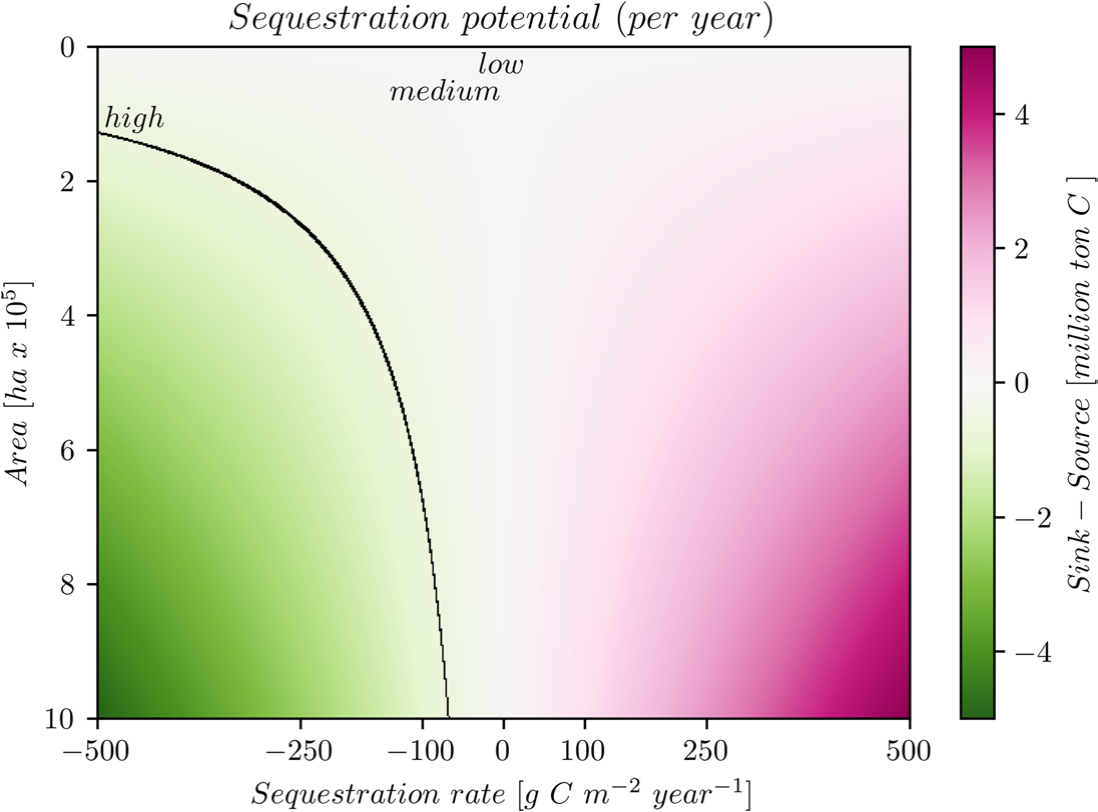
Sequestration potential [million ton C year] of Swedish farmlands illustrating the annual sink as a function of area available (vertical axis) for management options promoting SOC sequestration and sequestration rate (horizontal axis). Negative values denote fluxes from the atmosphere to the soil and are hence sinks of C. The three scenarios (low, 30, medium 100, and high, 500 [g C m^−2^ year^−1^]) yield sequestration potentials of 0.003, 0.05 and 0.5 million ton (million ton = Tg) C per year. The current average source from Swedish agriculture is 23 [g C m^−2^ year^−1^] (0.68 million to C / 2,982,800 ha), corresponding to the black curved line in the left part. The location of the labels (low, medium and high) corresponds to their values on the horizontal (sequestration rate) and vertical (area) axes.

**Fig. 8:**
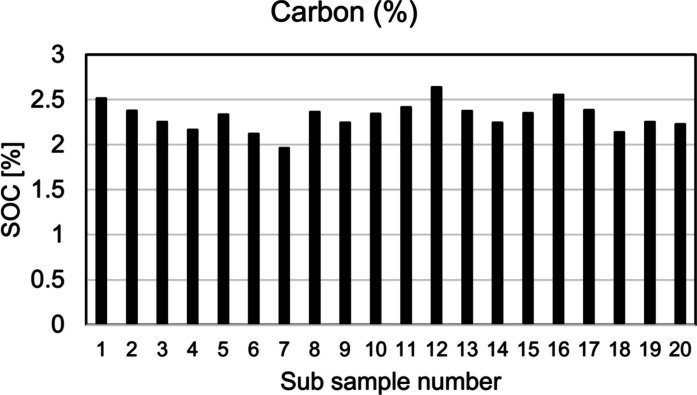
Soil Organic Carbon (SOC) for 20 subsamples taken out of sample 26 A illustrating the uncertainty originating from subsampling and from the instrument used for analysis of SOC

The total 2023 Swedish agricultural area was 2,982,800 ha, including 2,529,800 ha arable land (1,005,800 ha was cereals and 1,117,100 ha ley) and 453,000 ha pasture and hay meadow (Jordbruksverket, 2024). The 2023 average C source from Swedish agriculture was 23 g C m^−2^ year^−1^ (0.68 million to C/2,982,800 ha) (Jordbruksverket, 2024). Assuming the management based, annual sequestration potential of Swedish agricultural land to equal *rate* (g C m^−2^ year^−1^) × *area* (ha), the action space available for SOC sequestration can be illustrated (Fig. 7).

Our three (low, medium, high) sequestration scenarios (30, 100 and 500 g C m^−2^ year^−1^) × (10,000, 50,000 and 100,000 ha) yield sequestration potentials of 0.003, 0.05 and 0.5 million tons (1 million ton = 1 Tg) C per year. This corresponds to 0.2%, 2.9% and 28.8%, respectively, of the total CO_2_e emission from agriculture (6.44 million tons CO_2_e = 1.75 million tons C or 1.75 Tg C) (Jordbruksverket & Naturvårdsverket, 2022). These sequestration potentials correspond to 0.4%, 7.4% and 73.5% of the CO_2_e originating from *agricultural land* (i.e. not from animal metabolism and storage of manure) (Naturvårdsverket, 2024a).

Aiming for 10% annual reduction of the emissions from agricultural land, which corresponds to 68,000 ton C year^−1^, would require 230,000 ha, 70,000 ha and 14,000 ha respectively for the low, medium and high sequestration scenario. For the sequestration rates in our three scenarios (30, 100 and 500 g C m^−2^ year^−1^), this corresponds to 54 (35), 16 (10) and 3 (2) years of sequestration before the sequestration could be significantly detected (MDD method) with 10 (20) soil samples assuming *σ* = 2.94 mg C g soil^−1^.

The 4 per 1000 initiative (Soils for food security & climate, 2022) suggests an annual growth rate in soil carbon stocks of 0.4% (4 ‰) per year, in the upper 30–40 cm. For this soil with an initial C content of 7.95 kg C m^−2^ (Fig. 4, upper 30 cm), a consecutive 30-year sequestration of 0.4% would yield 1.01 kg C m^−2^ (10.1 Mg C ha ^−1^ year^−30^ or 34 g C m^−2^ year^−1^), i.e. more or less the same as the SCS standard sequestration rate (Svensk Kolinlagring, 2024). This sequestration rate requires 48 (31) years to provide a significant detectable difference (MDD) verified using 10 (20) samples.

From a broader perspective, extensive cultivation of low-productivity perennial crops would require food production to occur elsewhere to maintain overall output, thereby risking carbon leakage. Carbon sequestration is only one of several ecosystem services that need to be improved to support agricultural sustainability, and it will not always coincide with other desired ecosystem services, such as maintained food production. Nevertheless, recent developments in perennial crops (Zhang et al., 2023), together with their multidimensional environmental and other benefits (Chapman et al., 2022; Crews et al., 2018; Olsson et al., 2024; Soto-Gómez & Pérez-Rodríguez, 2022), suggest that this remains a worthwhile direction for research, particularly in combination with other pathways towards agricultural sustainability (Paustian et al., 2016). Thus, Fig. 7 illustrates *one dimension* of potential change, showing the potential of how investigation of perennial crop properties may support a transition in agriculture from a source to a sink of SOC, that is, moving from right to left in Fig. 7

## Conclusions

In situ measurements of SOC provide data allowing quantification of current levels as well as to infer SOC changes over time due to management activities increasing SOC. Applying statistical methods such as the MDD tends to require an unsuitable high number of soil samples to allow significant validation of changes over time, especially considering multisource variability (spatial, temporal, analytical). Such large samples may be justified when investigating SOC changes related to process understanding of mitigation efforts, but are not suitable for more general monitoring and verification of carbon credits. For monitoring, reporting and verification of carbon credit systems, alternate, more efficient methods are needed. Especially if an upscaling to considerably larger areas is to be implemented, potentially considering changing SOC stocks down to 1 or 2-m soil depth due to cultivation of perennial crops with deep roots. This is still an unsolved problem under development.

Viewed from this broader perspective, this study, based on in situ soil sampling, highlights the need for efficient and reliable MRV (Monitoring, Reporting and Verification) systems for soil carbon credit schemes. Recent work (Oldfield et al., 2022; Raffeld et al., 2024; Smith et al., 2020) emphasizes that credible soil carbon markets require consistent, transparent and robust approaches for quantifying soil organic carbon change and associated greenhouse gas impacts. This may include establishment of relationships between management change and SOC change, providing an activity-based proxy for SOC change at an environmentally comparable regional level (Smith et al., 2020). Standardized, nested regional frameworks that combine direct soil sampling, process-based modelling and remote sensing could improve comparability, reduce uncertainty and support more climatically reliable soil carbon crediting (Oldfield et al., 2022; Petropoulos et al., 2025; Smith et al., 2020).

## Data Availability

The results of the systematic SOC analysis (72 samples x 3 depths), the deeper soil samples (*n*=15) and the bulk density data is available in an excel file at https://zenodo.org/records/20703181. The data used for stratification is available as TIFF files from the corresponding author upon request.

## Appendices

### Sample representativity

For one randomly selected sample point (26 A), we analysed 20 subsamples (10–20 mg each) out the full sample which was around 500 g. Mean SOC was 2.31%, with a min of 1.96%, a max of 2.64% and a standard deviation of 0.16%. Relative standard deviation was 6.8%. This corresponds to a min of 8.8, mean of 10.4 and max of 11.9 (kg C m^−2^) for the upper 30 cm, yielding a 95% confidence interval for the mean of 2.24–2.38% SOC.
